# Sperm protein 17 is expressed in the sperm fibrous sheath

**DOI:** 10.1186/1479-5876-7-61

**Published:** 2009-07-15

**Authors:** Maurizio Chiriva-Internati, Nicoletta Gagliano, Elena Donetti, Francesco Costa, Fabio Grizzi, Barbara Franceschini, Elena Albani, Paolo E Levi-Setti, Magda Gioia, Marjorie Jenkins, Everardo Cobos, W Martin Kast

**Affiliations:** 1 Division of Hematology & Oncology, Texas Tech University Health Sciences Center Lubbock, TX, USA; 2 Southwest Cancer Treatment and Research Center, Texas Tech University Health Sciences Center Lubbock, TX, USA; 3 Kiromic, Inc Lubbock, TX, USA; 4 Department of Human Morphology, University of Milan, Milan, Italy; 5 Laboratories of Quantitative Medicine, Istituto Clinico Humanitas IRCCS, Rozzano, Milan, Italy; 6 Department of Reproductive Pathology, Istituto Clinico Humanitas, Rozzano, Milan, Italy; 7 Department of Internal Medicine, Texas Tech University Health Sciences Center, Amarillo, TX, USA; 8 Department of Obstetrics & Gynecology, Texas Tech University Health Sciences Center, Amarillo, TX, USA; 9 Laura W Bush Institute for Women's Health and Center for Women's Health and Gender-Based Medicine, Texas Tech University Health Sciences Center, Amarillo, TX, USA; 10 Department of Molecular Microbiology & Immunology, Norris Comprehensive Cancer Center, University of Southern California, Los Angeles, CA, USA; 11 Department of Obstetrics & Gynecology, Norris Comprehensive Cancer Center, University of Southern California, Los Angeles, CA, USA; 12 Cancer Research Center of Hawaii, University of Hawaii at Manao, Honolulu, Hawaii, USA

## Abstract

**Background:**

Sperm protein 17 (Sp17) is a highly conserved mammalian protein characterized in rabbit, mouse, monkey, baboon, macaque, human testis and spermatozoa. mRNA encoding Sp17 has been detected in a range of murine and human somatic tissues. It was also recognized in two myeloma cell lines and in neoplastic cells from patients with multiple myeloma and ovarian carcinoma. These data all indicate that Sp17 is widely distributed in humans, expressed not only in germinal cells and in a variety of somatic tissues, but also in neoplastic cells of unrelated origin.

**Methods:**

Sp17 expression was analyzed by immunocytochemistry and transmission electron microscopy on spermatozoa.

**Results:**

Here, we demonstrate the ultrastructural localization of human Sp17 throughout the spermatozoa flagellar fibrous sheath, and its presence in spermatozoa during in vitro states from their ejaculation to the oocyte fertilization.

**Conclusion:**

These findings suggest a possible role of Sp17 in regulating sperm maturation, capacitation, acrosomal reaction and interactions with the oocyte zona pellucida during the fertilization process. Further, the high degree of sequence conservation throughout its N-terminal half, and the presence of an A-kinase anchoring protein (AKAP)-binding motif within this region, suggest that Sp17 might play a regulatory role in a protein kinase A-independent AKAP complex in both germinal and somatic cells.

## Background

The interaction of capacitated spermatozoa with the zona pellucida (ZP) of the oocyte is a complex process involving a high number of spermatic molecules [1]. A family of low molecular weight sperm autoantigens has been recognized in rabbit spermatogenic cells and mature spermatozoa, and shown to bind the carbohydrate components of the ZP[2]. Among them, a mannose-binding protein called Sperm protein 17 (Sp17) was firstly isolated, sequenced and characterised in rabbit [3] and subsequently in a wide range of mammalian species [4-7]. As in rabbit and mouse [4], human Sp17 has been identified to be an autoantigen in sera from both vasectomized and vasostomized men [7]. Although Sp17 was originally thought to be gamete-specific, mRNA encoding Sp17 has been found in a range of murine [8] and human somatic tissues [9]. It was also detected in two myeloma cell lines [10] and in neoplastic cells from patients with multiple myeloma and ovarian carcinoma [11,12]. Using self-produced mouse anti-human Sp17 antibodies, this protein was recognized in the cytoplasm of some spermatocytes and that of early and late spermatids [13]. The flagella of the spermatozoa in the lumen of the seminiferous tubules was also found to be immunopositive for Sp17. Recently, Sp17 was found expressed in the synoviocytes of females affected by rheumatoid arthritis [14], human ciliated epithelia [15] and the melanophages of cutaneous melanocytic lesions [16].

All these data demonstrate that Sp17 is more widely distributed in humans than originally thought, expressed not only in germinal cells and in a variety of somatic tissues, but also in neoplastic cells of unrelated histological origin. For this reason, the definition of the biological role of Sp17 remains an open question.

The present study was aimed at investigating the localization of Sp17 by morphological methods during *in vitro *states of the dynamical process that spermatozoa go through from sperm ejaculation to the oocytes fertilization, since currently we do not know whether human Sp17 is localized on the surface of the spermatozoa, after sperm-ZP contact occurs. In addition, we aimed at analyzing the ultra-structural localization of Sp17 in human spermatozoa by electron microscopy in order to provide new information useful in understanding the biological function of Sp17.

## Methods

### Samples

The study, using human subjects, was carried out in accordance with the guidelines of the Ethics Committee of the hospitals involved.

Spermatozoa collected by ejaculation within a minimum of 48 hours but not longer than seven days of sexual abstinence from 26 fertile donors were analyzed. Motile and morphologically normal spermatozoa were selected using a discontinuous PureSperm gradient (Nidacon Laboratoires, AB, Gothenburg, Sweden), and some were incubated at 37°C for 45 minutes in a hypo-osmotic medium in order to assess the functional integrity of the sperm plasma membrane.

To evaluate the expression of Sp17 in ZP-bounded spermatozoa, ZPs were collected from empting immature non-inseminated oocytes or non-fertilized oocytes after intracytoplasmic sperm injection (ICSI). Each ZP was incubated overnight with treated semen and then removed and pipetted in order to dislodge loosely attached spermatozoa. Sperm collection, viability, hypo-osmotic swelling test and ZP binding were performed in accordance with the instructions in the WHO laboratory manual for the examination of human semen and sperm-cervical mucus interaction [17].

### Immunocytochemical analysis

To investigate the immunocytochemical expression of Sp17, spermatozoa were fixed with Biofix (Bio-Optica, Milan, Italy), and subsequently permeabilized with 0.5% Triton X-100 (Sigma Ltd, Missouri, USA); 0.1% sodium citrate in phosphate buffered saline, PBS) at 4°C for 15 minutes, followed by treatment with either primary mouse antibodies raised against human Sp17 [13-15] at room temperature for two hours, or with 1 μg/ml mouse IgG1 (Dako, Milan, Italy) as a negative control. This was followed by 30 minutes incubation with the DAKO Envision System (Dako, Milan, Italy). 3, 3'-diaminobenzidine tetrahydrochloride (Sigma Ltd, Missouri, USA, 12.5 mg and 500 μl H_2_O_2 _in 50 ml of TRIS buffer saline) was used as a chromogen to yield brown reaction products. Almost one thousand spermatozoa were counted under a light microscopy (Leica DMLA, Milan, Italy) and the number of those immunopositive for Sp17 was expressed as the mean percent ± SD of all spermatozoa.

### Transmission electron microscopy

Sperm pellets were centrifuged twice at 1100 rpm for 10 minutes and samples were fixed 4 hours either in 3% glutaraldehyde in PBS to perform ultrastructural analysis or in 4% formaldehyde in PBS pH 7.4 at 4°C to evaluate Sp17 ultra-structural localization. All cell pellets were then washed with PBS. For ultrastructural analysis, cells were post-fixed in 1% osmium tetroxide (OsO_4_) in 0.1 M PBS, dehydrated through an ascending series of aceton, and embedded in Durcupan (Fluka). Ultrathin sections were obtained with an Ultracut ultramicrotome (Reichert-Jung), stained with uranyl acetate and lead citrate before examination by a Jeol CX100 electron microscope (Jeol, Tokyo, Japan).

Formaldehyde-fixed cells were immersed in NH_4_Cl 0.25 M in PBS overnight at 4°C and embedded in Durcupan (Fluka) K4M After washing, samples were dehydrated in ascending concentrations of cold ethanol, embedded in Lowicryl K4M (Agar, Polysciences Inc., Stansted, UK) at -35°C and polymerized under UV light (360 nm) at -35°C for a week. Ultrathin sections were cut by a diamond knife with a Reichert Ultracut R ultramicrotome and collected on celloidine-coated 100 mesh nickel grids (Electron Microscopy Sciences, Società Italiana Chimici, Rome, Italy). For the immunogold procedure, sections were incubated at room temperature with 0.03% saponin in 0.05 M TBS/1% BSA (buffer A, pH 7.6) for 30 minutes and then autoclaved at 121°C in EDTA 1 mM (pH 8) for 15 minutes. After cooling, grids were incubated for 2 hours with mouse anti-human Sp17 antibodies [13-15]. After washing in buffer A, grids were incubated with a goat anti-mouse IgG (Aurion, Wageningen, The Netherlands) for 1 hour at room temperature. Sections were then washed and examined with a JEM 1010 transmission electron microscope (Jeol, Tokyo, Japan) at 80 kV.

## Results and discussion

**Figure 1:**
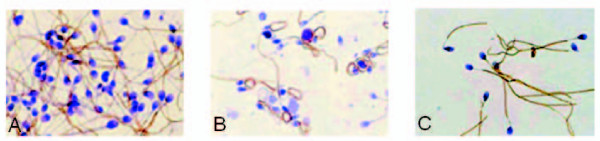
**Expression of Sp17 in human spermatozoa collected from fertile donors**. Immunohistochemistry demonstrates that Sp17 is localized throughout the principal piece of the flagellum. The expression of Sp17 is maintained in all of the three classes of investigated spermatozoa: density-gradient (A), swelling (B), and ZP-bound spermatozoa (C) Although, we observed a number of immunopositive spermatozoa was observed, ZP-bound spermatozoa show a more intense staining (C).

Two distinct Sp17 populations, one immunopositive (amounting for about 90% of all the spermatozoa) and the other immunonegative, were recognized in all of the three classes of investigated spermatozoa (density gradient, hypo-osmotic swelling, and ZP-bound spermatozoa). At higher magnification, Sp17 is clearly detectable throughout the principal piece of the flagellum, but the intermediate piece, (Figure 1B) head and acrosomal vesicle were always immunonegative (Figure 1A–C).

Although no differences were obtained comparing the mean percentages of immunopositive spermatozoa belonging to the three classes, an increased percentage of immunopositive spermatozoa and a marked immunoreactivity were evident in ZP-bound spermatozoa (Figure 1C). Interestingly, changes in the integrity and compliance of the sperm plasma membrane did not modify the presence of Sp17 (Figure 1B).

**Figure 2:**
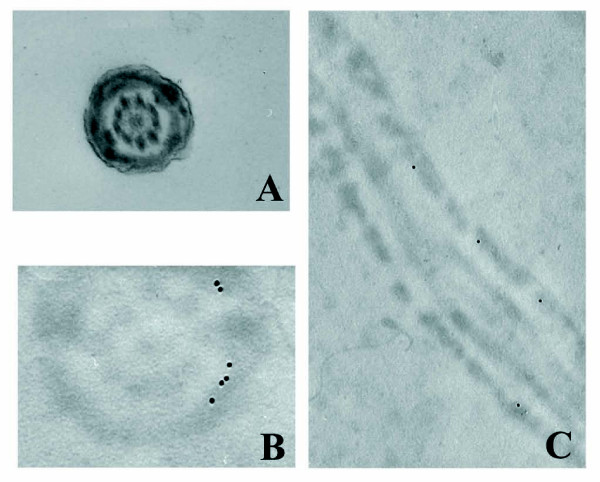
**Transmission electron microphotographs of human ejaculated spermatozoa collected from fertile donors**. A: Araldite transverse section; B and C: Sp17 immunogold labeling in Lowycril transverse (B) and longitudinal (C) sections. Immunoreactivity was restricted to the fibrous sheath and no gold particles were localized in the axonema or in outer dense fibers. Original magnification: A: 29000; B and C: 25000×.

Ultra-structural immunochemistry completely confirmed light microscopy observations, clearly demonstrating that spermatozoa expressed the Sp17 protein and that the epitope recognized by Sp17 antibodies was localized throughout the principal piece of the flagellum. Transverse sections showed that immunoreactivity was restricted to the fibrous sheath (FS) and no gold particles were localized in the axoneme or in outer dense fibers (Figure 2A). In longitudinal sections, Sp17 immuno-labelling was more intense in the inner than in the outer FS (Figure 2B).

The expression of Sp17 was previously demonstrated in the differently differentiated stages of spermatozoa maturation such as in spermatocytes, spermatids and spermatozoa [13], and its marked developmentally regulated increase in testis indicated that Sp17 plays an important role in sperm function [9]. Moreover, the present findings showed Sp17 immunoreaction with ZP of the oocyte. These overall findings suggest a role of Sp17 during sperm maturation, capacitation, acrosomal reaction and the fertilization process.

In addition, the wide distribution in germinal, somatic and tumoral cells indicates that the proposed role of Sp17 in ZP binding is unlikely to be its unique function as originally thought. In fact, immunohistochemical studies showed that Sp17 is expressed not only in germinal tissues, but also in the human respiratory airways and reproductive systems, and in particular in ciliated cells [15].

The definition of Sp17 functional role is still an open question. A re-evaluation of Sp17 has been recently introduced by Frayne and Hall [9]. In an attempt to discover a possible function of the extremely conserved N-terminal domain of Sp17, they noticed that this region contains a motif (first 74 aminoacids) that is very similar to the N-terminal sequence of the cAMP-dependent protein kinase A regulatory subunit II (PKA RII), which is essential for protein dimerisation and interaction with A-kinase anchoring proteins (AKAPs). It is plausible that Sp17 plays a regulatory role in a PKA-independent AKAP complex in both somatic and germinal cells. AKAPs represent a family of sequence-unrelated proteins classified exclusively by their ability to bind PKA *in vitro*, and possess targeting domains that mediate their attachment to the plasma membrane, cytoskeleton, or intracellular organelles [18]. Furthermore, AKAPs bind simultaneously to PKA and other signal transduction molecules, leading to the hypothesis that their function is related to the coordination of several signalling proteins [19-21].

Interestingly, it was demonstrated that Sp17 and AKAP3 are specifically associated in spermatozoa flagella [22]; since AKAP3 acts as a scaffold protein in binding various components of signal transduction pathways, this evidence may be relevant in understanding the functional role of Sp17, and in particular, in relation to motility. Actually, three sperm-specific AKAP-binding proteins have been identified, namely ropporin [23], AKAP-associated sperm protein [24] and fibrousheathin II [25]. These three proteins have been localized to the FS of the sperm tail. Analogously, the present study is the first to demonstrate by electron microscopy that Sp17 is a FS protein. The FS is a unique cytoskeletal structure surrounding the axoneme and outer dense fibers and defines the extent of the principal region of the sperm flagellum. Despite a number of proteins present in the FS having been identified [26], there is little experimental evidence shedding light on the possible function of this sperm structure. Interestingly, Kultgen et al [27] have recently provided the first biochemical data showing that a pool of PKA was also localized within human ciliary axonemes. Additionally, they have identified the first human AKAP targeted to the ciliary axoneme, named AKAP28 in cilia of columnar cells of the respiratory airways, but not in goblet and basal cells, and proposed that AKAP28 localizes PKA to a position in the axoneme where it is able to readily interact with its substrate. Similar results were obtained in hamster oviducts by Morales et al [28]. These data reinforce the idea that Sp17 might play an important role in cell signalling in both somatic and germinal cells, as well as in cell motility.

Sp17 was demonstrated to be highly expressed in tumor tissues and cells [10-12,29]. A possible role of Sp17 was demonstrated in transformed lymphoid and hematopoietic cells [8], suggesting that Sp17 may be involved in tumorigenesis mechanisms by its ability to mediate cell adhesion and interaction, and therefore migration of malignant cells [29].

## Conclusion

In conclusion, the present study designates Sp17 as a novel FS protein and shows the continuous presence of this protein throughout the sperm tail from ejaculated spermatozoa to the sperm-ZP binding phase. These data contribute to the understanding of Sp17's biological role and its possible clinical implications.

## Abbreviations

AKAPs: kinase anchoring proteins; PKA: protein kinase A; Sp17: sperm protein 17; ZP: zona pellucida.

## Competing interests

The authors declare that they have no competing interests.

## Authors' contributions

MCI carried out the study design, drafted the manuscript and coordination and revised the manuscript and is responsible of some of the sperm collection and viability analysis.

NG participated in the design of the study, and revised the manuscript.

FG participated in the design of the study, and revised the manuscript.

ED performed electron microscope analysis.

FC performed immunohistochemical analysis.

BF performed immunohistochemical analysis.

EA is responsible of sperm collection, viability analysis, hypo-osmotic swelling test and zona pellucida binding.

PLS revised the manuscript.

MG revised the manuscript.

MJ participated in study design and coordination, and revised the manuscript.

EC participated in study design and coordination and revised the manuscript.

WMK participated in study design and coordination and revised and drafted the manuscript.

All authors read and approved the final manuscript.
